# Short-Term Flexural Stiffness Prediction of CFRP Bars Reinforced Coral Concrete Beams

**DOI:** 10.3390/ma14020467

**Published:** 2021-01-19

**Authors:** Lei Wang, Jin Yi, Jiwang Zhang, Wu Chen, Feng Fu

**Affiliations:** 1 College of Civil Engineering and Architecture, Guilin University of Technology, Guilin 541004, China; wanglei@glut.edu.cn (L.W.); 6616056@glut.edu.cn (J.Y.); zhangjiwang@glut.edu.cn (J.Z.); 2 Guangxi Key Laboratory of Geomechanics and Geotechnical Engineering, Guilin 541004, China; 2017040@glut.edu.cn; 3 School of Mathematics, Computer Science and Engineering, City, University of London, London EC1V 0HU, UK

**Keywords:** coral concrete beams, CFRP bars, strain inhomogeneity coefficient, flexural stiffness, relative slip

## Abstract

FRP (Fiber Reinforced Polymer) Bar reinforced coral concrete beam is a new type of structural member that has been used more and more widely in marine engineering in recent years. In order to study and predict the flexural performance of CFRP reinforced coral concrete beams, the flexural rigidity, crack morphology and failure mode of concrete were studied in detail. The results show that under the condition of similar reinforcement ratio, the flexural rigidity of CFRP reinforced coral concrete beam is significantly lower than that of ordinary reinforced concrete beam. Increasing the cross-section reinforcement ratio within a certain range can increase the bending stiffness of the test beam or reduce the deflection, but the strength utilization rate of CFRP reinforcement is greatly reduced. The short-term bending stiffness of the CFRP reinforced coral concrete beam calculated by the existing standard formula is obviously higher. This paper proposes a modified formula for introducing the strain inhomogeneity coefficient (ψ) of CFRP bars and considers the relative slip between CFRP bars and coral concrete to predict the short-term flexural stiffness of coral concrete beams reinforced by CFRP bars. The formula was verified with the test results, and it was proved that the formula has a good consistency with the test results.

## 1. Introduction

Recently, coral concrete has been gradually used in the coastal area and islands for building construction due to its environment friendly feature. coral concrete with the use of coral reefs and other island reef deposits as aggregates and seawater instead of freshwater can greatly reduce the production and transportation costs. It is major composition, coral debris, can be easily found from the ocean. This new type of product therefore has a high economic value without damaging the natural environment. However, corrosion of reinforcing bars caused by the humidity and hot marine environment in the coastal area and the large amount of salt in coral debris cause the main durability problem of coral concrete [1,2]. Fiber Reinforced Polymer Bar (FRP Bar) has been widely used due to its excellent mechanical properties and excellent corrosion resistance, which provides a new solution for the durability of coral concrete structures [3,4,5]. However, due to the unique composition of coral concrete material, the research on FRP reinforced coral concrete structure is rare. The structural behavior and performance of FRP reinforced coral concrete structure are not fully understood.

FRP reinforced concrete beams are characterized by large deflection, wide crack width and no obvious yield stage [6,7,8,9]. Gdoutos et al. [10] pointed out that the lower elastic modulus and unique bonding characteristics of FRP bars are the main reasons for the larger deformation of FRP bars reinforced concrete members than that of reinforced concrete members with the same concrete strength, load, component size and reinforcement ratio. Sandor et al. [11,12] showed that the direction of the radial force component of FRP bars in concrete is quite different from that of steel bars, and the FRP bar specimens are more prone to split failure. Zhu et al. [13] discovered that short-term flexural rigidity of FRP bars reinforced concrete beams is easy to be overestimated because the potential influence of bond-slip of FRP bars is not taken into account. Wang et al. [14] compared the calculation results of 745 crack data with different national codes and found that the existing codes overestimated the crack width of FRP reinforced coral concrete beams, which affected the evaluation of the flexural performance of the members. Issa et al. [15] suggest that the deformability of FRP reinforced concrete beams need to be checked. Gravina et al. [16] predict the flexural capacity of FRP reinforced concrete beams with the consideration of the spacing and width of flexural cracks, and the bonding performance between FRP bars. Raed et al. [17] believe that the assumption of plane section remain plane in the calculation of deflection of FRP reinforced concrete beams and slabs under high-level load will lead to a significant reduction in the rigidity of FRP reinforced concrete members, resulting in significant deflection. Wang et al. [18] found that the flexural performance of concrete beams of different types of reinforcement is significantly different, and the application of FRP reinforcement to different types of concrete is also different. The existing calculation model is difficult to accurately predict their flexural performance. Sun et al. [19] pointed out that the ultimate displacement of FRP bars test beam is 1.6–1.9 times greater than that of steel bars test beam due to the difference in bond behavior. Xu et al. [13,20] analyzed different calculation models based on a large number of experimental data and deduced the calculation formulas. The results show that the existing calculation models are still ignored the unique feature of high tensile strength, low elastic modulus, brittle failure mode and low bond performance of this unique type of structure. Therefore, there are inaccurate to predict the short-term stiffness for this particular type of structure. 

In order to understand the changing characteristics of the flexural performance of the test beam under different loads, accurately calculate and predict the flexural rigidity of the FRP bar-reinforced coral concrete beam, and establish a calculation model for the flexural stiffness, the flexural behavior, short-term stiffness and failure modes of CFRP reinforced coral concrete beams are studied in detail through full scale flexural tests.

## 2. Test Program 

Six coral-concrete beams reinforced with fiber bars and two coral-concrete beams reinforced with steel were tested by four-point bending test. All test beams were designed according to existing fiber-reinforced concrete codes and were load-graded using force control. Coral concrete is a kind of lightweight aggregate concrete.

### 2.1. Test Specimens 

**Figure 1:**
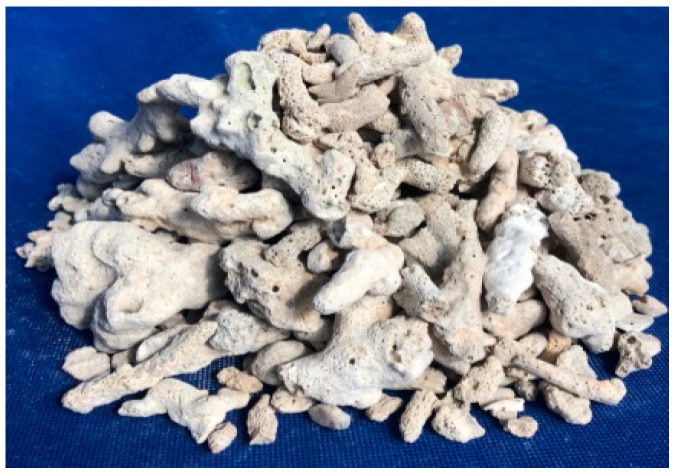
Coral debris.

The CFRP bars used in the tests were manufactured by Zhejiang Haining Anjie Composite Material Co., Ltd. (Haining, Zhejiang, China). Their basic mechanical properties of shown in Table 1. The cement is P.042.5 ordinary Portland cement produced in Xing’an, Guangxi. The coarse aggregate is natural continuous graded coral debris (Figure 1) from North Harbor of China South Sea. The fine aggregate is made of ordinary natural river sand. The water is artificial seawater and polycarboxylic acid superplasticizer is added. At the same time, six cubic and six prism concrete specimens were casted and tested after 28 days of curing under the same conditions as the test beams. The axial compressive strength, tensile spitting strength and elastic modulus of coral concrete were measured. The mix ratio and basic mechanical parameters of coral concrete were shown in Table 2.

**Figure 2:**
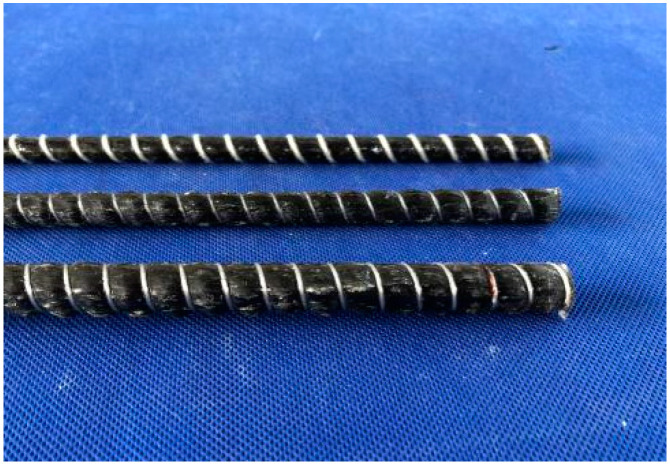
CFRP bars.

Eight beams were fabricated with the length of 2400 mm. Their net span is 2100 mm. The sectional dimension is 120 × 250 (mm^2^), the cover of the concrete is 25 mm, and the diameters of the longitudinal bars are 8 mm, 10 mm and 12 mm (Figure 2), respectively. The basic parameters are shown in Table 3. 

### 2.2. Instrumentation and Loading Process

**Figure 3:**
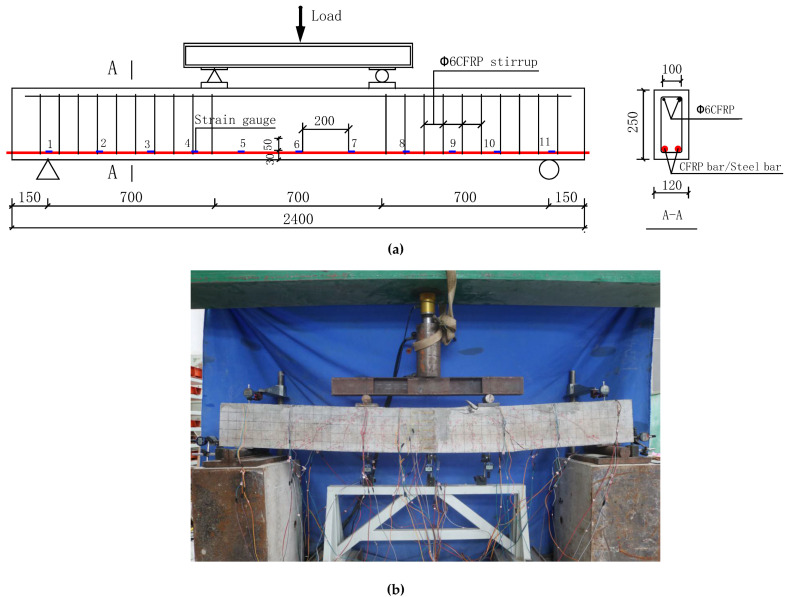
(**a**) Test specimen: geometry and reinforcement detail (dimensions in mm); (**b**) Actual loading condition of test beam.

Similar to test set up in [21,22,23,24,25], as it shown in Figure 3a,b, strain gauges are symmetrically arranged at 200 mm intervals on the CFRP bars and five strain gauges (BA120-05AA) are pasted at equal intervals on the front and back surface of concrete in the middle of the span. All strain gauges are connected to a strain tester (DONGHUA DH3816N, Jingjiang, Jiangsu, China) for measurement. It can be also seen that dial gauges are placed at the midspan, loading point and support of the beam respectively to measure the deformations at these positions. In addition, dial gauges are placed at the end of the longitudinal reinforcement at beam end to measure the end slip of the beams. 

Four-point bending is adopted, which is loaded by a reaction frame and a hydraulic jack (PATELI YEYA QF50T-20k, Hongkou, Shanghai, China) with a load transducer (HUADONG ELEC-TECH BHR-4 30T, Jingan, Shanghai, China). During the tests, the load is increased with 3 kN increment at each step. Each loading step is held for 5 min to ensure the instrumentation data is stabilized and the test data are recorded before the load was continuously to increase until the specimen is destroyed. In addition, the electronic crack monitor device (ZBL-F103, Xicheng, Beijing, China) is used to measure the crack width of the concrete at the height of the longitudinal rebars, and the crack development height and crack development speed are measured and recorded. 

## 3. Test Result 

According to the test results, the bending performance of the test beams with different reinforcements is analyzed and compared. The development rate of deflection and crack width of the fiber reinforced test beam at different stages is different from that of the reinforced test beam. The fiber reinforcement test beam has problems such as large crack width, relative slippage of the reinforcement, and low utilization rate of the reinforcement. The stiffness changes under different load levels are worthy of further discussion.

### 3.1. Load Midspan Deflection Relationship 

**Figure 4:**
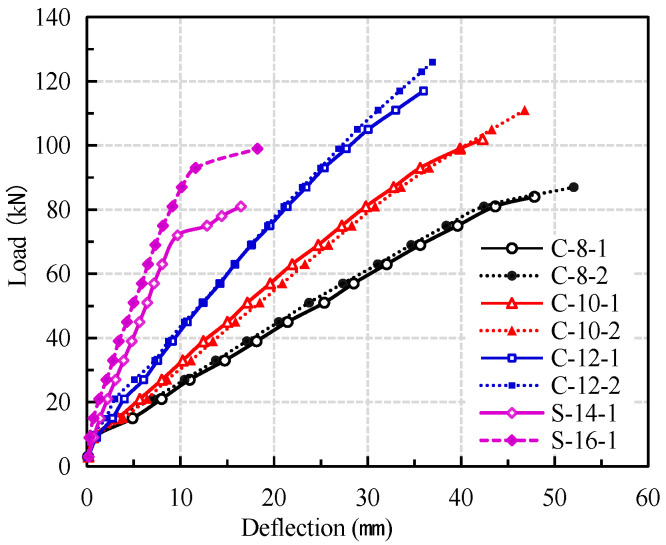
Effect of reinforcement ratio of CFRP bars on load-deflection curves.

From the load-midspan deflection curve of the beams in Figure 4, it can be seen that the mid-span deflection of the beams increased with the increase of load, but the deflection curves of the beams with different type of reinforcement and different reinforcement ratios are significantly different. The load-deflection curves of CFRP reinforced coral concrete beams are not significantly different from those of conventional reinforced concrete beams. The conventional reinforced concrete beams can be roughly divided into three working stages: pre-cracking, post-cracking elasticity and reinforcement yielding. Since CFRP bar is a linear elastic material, the load-deflection curve of CFRP reinforced coral concrete beams can be divided into two working stages: no obvious yield stage and relatively sudden failure.

Before concrete crack formed in tension zone, the increase of deflection is slower with the increase of load. After concrete cracking, the stiffness of CFRP reinforced coral concrete beams decreased rapidly, and the increase rate of deflection is obviously accelerated. Under the same load conditions, the deflection of CFRP reinforced coral concrete beams is obviously larger than that of reinforced coral concrete beams. The ultimate bearing capacity of C-8 test beams is not much different from that of steel reinforced coral concrete beams, but the mid-span deflection of CFRP reinforced coral concrete beams under the same load is about 3–4 times of that of latter. Within a certain range, increasing the reinforcement ratio of CFRP bars can significantly improve the flexural rigidity and reduce the deflection. The maximum deflection of C-10 and C-12 beams is 10.8% and 27% lower than that of C-8 beams, respectively. However, with the increase of CFRP reinforcement ratio, the low strength utilization of FRP bars becomes more serious. According to GB 50608-2010 [26], the maximum deflection of l_0_/200 (l_0_ is the net span of the test beam) is allowed. When the midspan deflection of CFRP reinforced coral concrete beams reach l_0_/200 (10.5 mm), the load withstood by the CFRP reinforced coral concrete beam is only 23.5–38.5% of their ultimate bearing capacity. Under the same deflection, the steel reinforced coral concrete beams bear about 90% of their ultimate load. The test results are shown in Table 4.

### 3.2. Crack Width

**Figure 5:**
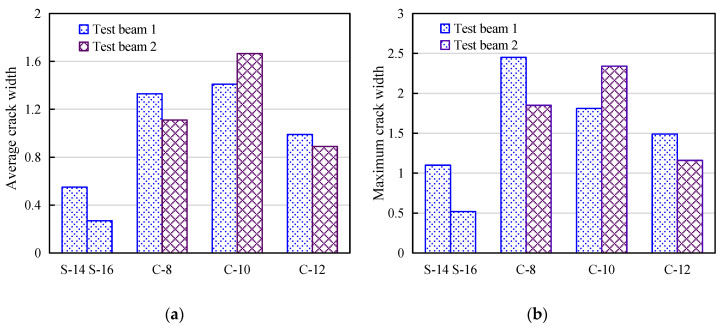
Comparison of crack width of specimen beams: (**a**) Average Crack width; (**b**) Maximum Crack width.

From Figure 5, it can be seen that under the same load conditions, the crack width of the CFRP bar reinforced beam is obviously larger than that of the steel reinforced beam, and the crack propagation height is also higher than that of the reinforced concrete beam. The average crack height is 40 mm for steel reinforced beam, and the average crack height accounts for 70% of the beam height when it reaches ultimate capacity, while the average crack height of CFRP bar reinforced beam is 140 mm, and the maximum crack height accounts for more than 90% of the beam height. The main reason is that after cracking of the concrete in the tension zone at the bottom of the test beam, the tensile stress at the cracks will be borne by the steel bar or CFRP bar. Because the elastic modulus of CFRP bar is obviously smaller than that of the steel bar, the bonding performance between CFRP bar and concrete is relatively weak [27,28,29,30,31], incurs larger deflection. With the increase of reinforcement ratio of CFRP bars, the crack development speed is slowed down, the average crack width and maximum crack width are obviously reduced, but the influence on crack spacing is not obvious. The number of cracks in each beam is between 11 and 15. It should be noted that the maximum crack width and average crack width of all test beams exceed the maximum width limit of 0.5 mm stipulated by current code [26]. 

### 3.3. Load and Strain Relation for Longitudinal Bar

**Figure 6:**
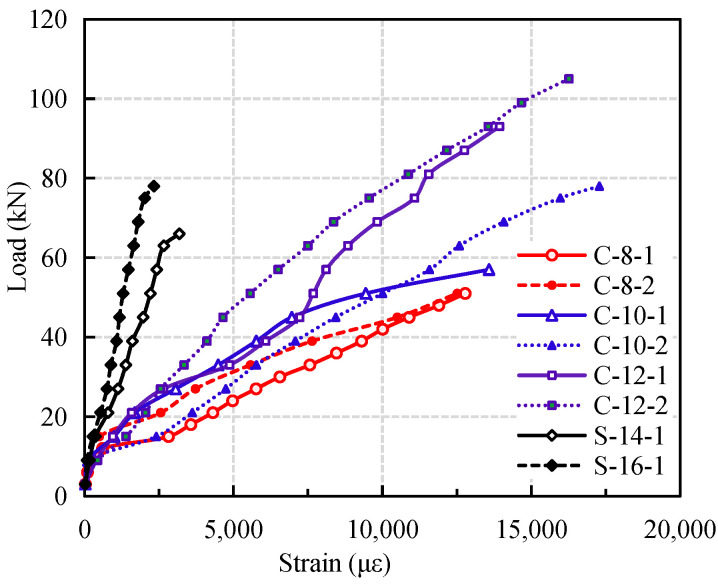
Effect of reinforcement ratio of CFRP bars on strain curve.

The development trend of load-strain curve of CFRP reinforced coral concrete beams is similar to that of ordinary reinforced concrete beams [11,12,13,14]. For ordinary reinforced concrete beams, it can be divided into three stages: slow growth of strain before cracking, uniform growth of longitudinal reinforcement after cracking, yield of reinforcement to failure, as shown in Figure 6. The load-strain curve of CFRP reinforced coral concrete beams can be divided into two stages: pre-cracking stage and post-cracking stage [7,8,9]. 

Before the concrete cracks in the bottom tension zone, the strain values of steel reinforced coral concrete beams and CFRP reinforced coral concrete beams are similar because concrete withstand more tension stress. After cracking, the strain of CFRP bars increases sharply with the increase of load, which is obviously larger than that of steel bars under the same load value. The main reason is that the bond strength between CFRP bars and coral concrete is weaker than that of reinforcing bars. The relative slip between CFRP bars and coral concrete results in a greater redistribution of the tensile stress of CFRP bars. With the increase of reinforcement ratio of CFRP bars, the slope of load-strain curve increases, and the strain of CFRP bars decreases under the same load. When the midspan deflection of CFRP reinforced coral concrete beams reaches *l*_0_/200 (10.5 mm), the strain of CFRP rebars under tension is about 30–41.9% of their ultimate strain, which indicates that the strength utilization ratio of CFRP bars is low. In addition, bond slip makes the strain of CFRP bar increase nonlinearly. 

### 3.4. Bond Slip of CFRP Reinforced Coral Concrete 

**Figure 7:**
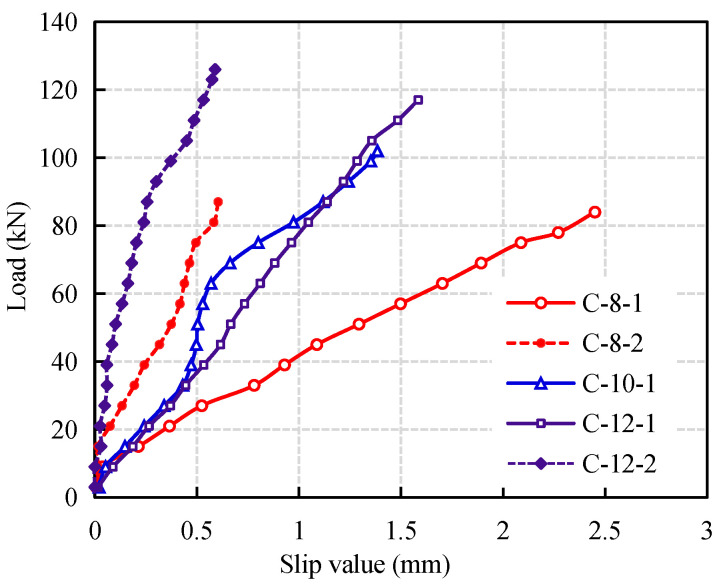
Relative slip curve of CFRP bars and coral concrete.

It should be noted that almost all CFRP bars reinforced coral concrete beams have slip between CFRP bars and coral concrete in varying degrees. The larger the load, the more obvious the slip. The relative slip curve of CFRP bars at the end of the test beams is shown in Figure 7 (There is no obvious slip in test beam C-10-2). It can be seen that the smaller the diameter of CFRP bars is, the larger the slip is. The slip of CFRP bars has a great influence on the flexural behavior of coral concrete beams. For the same reinforced beams, the flexural capacity of C-8-1 with larger slip is 3.6% lower than that of C-8-2, C-10-1 is 8.8% lower than that of C-10-2, and C-12-1 is 7.7% lower than that of C-12-2. It shows that the larger the diameter of CFRP bars, the smaller flexural capacity due to the slip. However, the chloride ions in artificial seawater and coral have no obvious effect on the surface of CFRP bars.

### 3.5. Failure Mode of the Beams 

**Figure 8:**
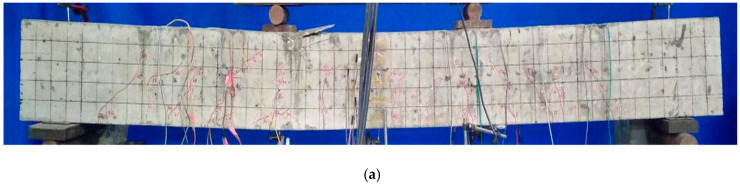

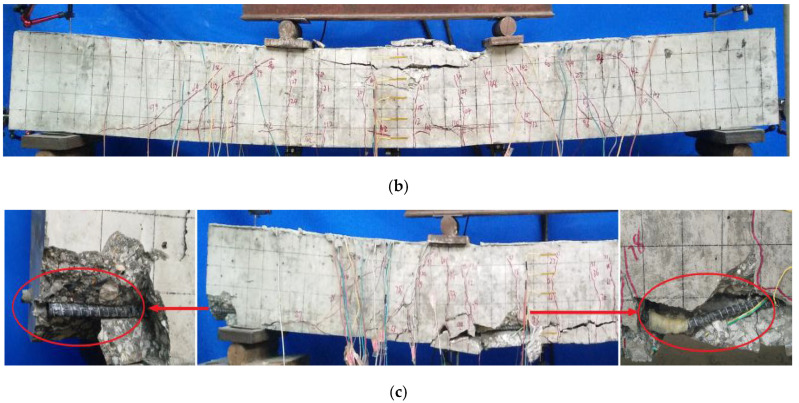
Failure pattern of specimen beams: (**a**) S-16-1 concrete crashing failure; (**b**) C-12-2 concrete crashing failure; (**c**) C-10-1 CFRP bar slip failure.

The failure modes of CFRP reinforced coral concrete beams are significantly different from those of steel reinforced coral concrete beams due to the different properties of reinforcement materials. The crack width and deflection of CFRP reinforced coral concrete beams are obviously larger than that of steel reinforced coral concrete beams. In addition, CFRP reinforced coral concrete beams have a large number of deep cracks along the longitudinal reinforcement direction. The slip between CFRP bars and coral concrete is also very serious. As shown in Figure 8, when the beam C-10-1 is damaged, the CFRP bars was exposed at the ends of the beams for about 16 mm. There is no slip phenomenon in steel reinforced coral concrete beams and obvious cracks along longitudinal reinforcement, which indicates that good bonding performance between reinforcement and coral concrete is maintained. However, when the deflection of the test beam increases, the concrete in the compression zone is damaged more obviously due to stress concentration. And when the CFRP bar and coral concrete slip locally, the radial component of the FRP bar causes the concrete protective layer to crack or peel off along the longitudinal direction of the bar. Moreover, the brittleness of coral concrete is greater than that of ordinary concrete, the concrete in the upper compressive zone of the middle-span interface of the test beam has a larger compressive damage range and obvious longitudinal cracks along the test beam, as shown in Figure 8a,b.

**Figure 9:**
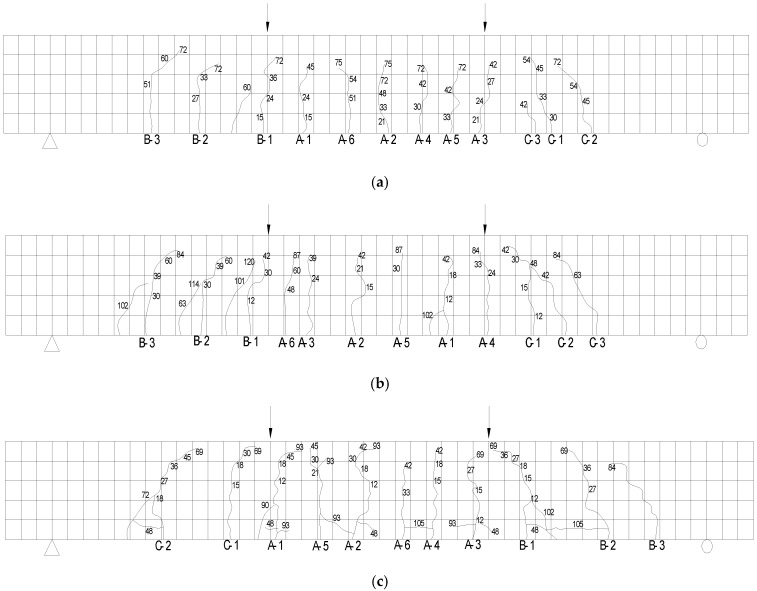

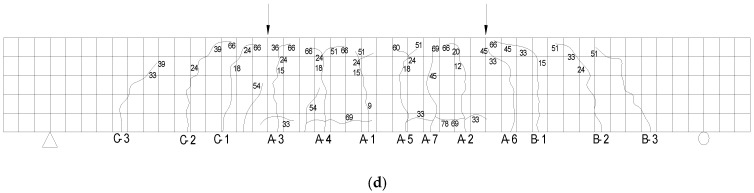
Crack distribution and development of the test beams: (**a**) S-16-1; (**b**) C-12-2; (**c**) C-10-1; (**d**) C-8-2.

Due to the slip of CFRP bars has a significant effect on the deflection and stiffness calculation of test beams, the relatively weak bond between FRP bars and concrete makes the strain of CFRP bars more uniform than that of steel bars [28]. When calculating flexural stiffness, the effect of relative slip of CFRP bars and coral concrete on stiffness reduction should be fully considered. In addition, the crack distribution of the test beam is shown in Figure 9.

## 4. Existing Formula for Short Term Flexural Rigidity 

This section mainly compares some standard calculation formulas or calculation models with the test results and discusses the differences between different calculation formulas and cause analysis.

### 4.1. Short Term Flexural Rigidity Formula

Because there are few studies on the flexural behavior of FRP reinforced coral concrete, therefore, no corresponding formula for calculating flexural stiffness. There is some formula for FRP bar reinforced normal concrete can be referred to. They are from ACI 440.1R-15 [32], Zhu Hong [13] and Chinese FRP design code [26].

#### 4.1.1. Formula from ACI 440.1R-15 (ACI Committee 440 2015)

The stiffness calculation formula of ACI 440.1R-15 is based on the code ACI 440.1R-06 [33] and ACI 318-05 [34]. Considering the load and boundary conditions and accounts for the length of the uncracked regions of the member and for the change in stiffness in the cracked regions. the stiffness factor γ is introduced in calculating the effective moment of inertia. It is as follows:

(1) $$I_{e}=\frac{I_{cr}}{1-\gamma {\left(\frac{M_{cr}}{M_{a}}\right)}^{2}[1-\frac{I_{cr}}{I_{g}}]}\leq I_{g}$$

(2) $$\gamma =1.72-0.72(M_{cr}/M_{a})$$

where *I_e_* is the effective moment of inertia of FRP bar reinforced beam after cracking, *M_cr_* is the moment when concrete cracks, *M_a_* is applied moment, *I_g_* is the moment of inertia of uncracked section, *I_cr_* is the moment of inertia of uncracked section, γ is the parameter to account for the variation in stiffness along the length of the member. $\beta_{d}$ is stiffness reduction factor.

#### 4.1.2. GB 50608-2010 (Chinese Standard 2011)

(3) $$B_{S}=\frac{E_{f}A_{f}h_{of}^{2}}{1.15\psi +0.2+\frac{6\alpha_{fE}\rho_{f}}{1+3.5\gamma_{f}^{\prime }}}$$

where, $B_{s}$ is the Test beam bending stiffness, ψ is strain inhomogeneity coefficient of CFRP bars, *E_f_* is the elastic modulus of fiber, *A_f_* is effective cross-sectional area, *h_of_* is the distance from the center of the longitudinal bar to the edge of the compression surface, $\alpha_{fE}$ is ratio of elastic modulus of fiber to concrete, $\rho_{f}$ is ratio of FRP bars in longitudinal tension: Considering $\rho_{f}=A_{f}/bh_{of}$, $\gamma_{f}^{\prime }$ is the ratio of area of section of compression flange to effective area of section of web.

#### 4.1.3. Zhu Hong (Hong et al. 2015)

Zhu [13] further modified Formula (3)

(4) $$B_{s}=\frac{E_{f}A_{f}h_{of}^{2}}{1.1\psi +0.2+\frac{6\alpha_{E}\rho_{f}}{1+3.5r_{f}^{\prime }}}$$

(5) $$\psi =1.3-0.74\frac{f_{tk}}{\rho_{te}\sigma_{fk}}$$

(6) $$\sigma_{fk}=\frac{M_{k}}{0.9A_{f}h_{of}}$$

where $f_{tk}$ is the tensile strength of concrete, $\sigma_{fk}$ is the tensile stress of longitudinal bars, $\rho_{te}$ is the ratio of longitudinal tensile FRP bars calculated according to the effective tensile concrete section area, $M_{k}$ is the bending moment calculated by standard combination of load effects. The other symbols have the same meaning as Formula (3).

### 4.2. Comparison to Test Results 

The formula for deflection is as follows:

(7) $$f=\frac{6.81Pl_{0}{}^{3}}{384E_{c}I_{e}}$$

where f is the midspan deflection, P is the load, $l_{0}$ is the clear span, $E_{c}$ is the Young’s module of coral concrete, $I_{e}$ is the moment of inertia of the section, $E_{c}I_{e}$ is the flexural rigidity of the beam.

**Figure 10:**
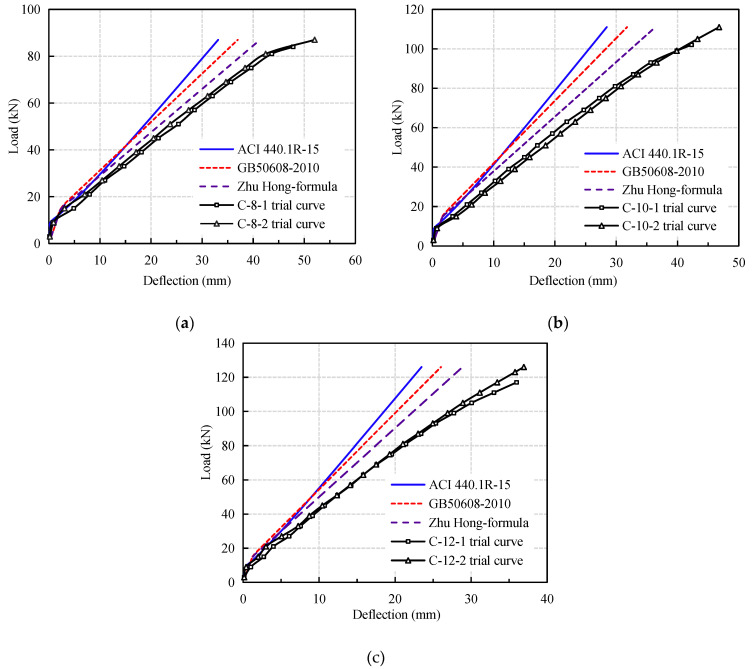
Comparison of calculated and experimental deflection of CFRP bars: (**a**) C-8; (**b**) C-10; (**c**) C-12.

From Figure 10, it can be seen that the short-term stiffness of CFRP reinforced coral concrete is obviously overestimated by the calculation formulas given by Chinese and American codes, and the difference of deflection calculation increases obviously with the increase of load. Among them, the effective moment of inertia calculation formula in ACI 440.1R-15 is modified on the basis of ACI 440.1R-06 and the stiffness reduction coefficient $\beta_{d}$ is not considered, which makes the pre-load deflection close to the calculated value, but with the increase of load, the deflection discrepancy becomes more serious. Although the calculation curve obtained by the modified formula proposed by Zhu Hong et al. [13] effectively reduces the error, the calculation value is still large when applied to the deflection calculation of coral concrete flexural members. There are two main reasons for the large discrepancy in the calculation. One is that the current formulas for calculating the stiffness of flexural members with FRP bars are mostly based on the formulas for design the normal reinforced concrete. There is not enough correlation between the elastic modulus of FRP bars and bonding performance to and that of steel reinforced concrete, this result in that the calculation are quite different to the actual result; secondly, the elastic modulus of coral concrete is lower than that of ordinary concrete of the same grade, and the bonding of FRP bars are relatively weak. The relative slip between FRP bars and coral concrete is larger. In addition, the compressive strength coral concrete in failure is higher than that of normal concrete. The brittleness of concrete is greater than that of ordinary concrete.

It should be noted that a large number of experimental studies show that the load-deflection curve of FRP reinforced concrete beams under bending is not an ideal linear relation, but presents certain non-linear characteristics, especially in the stage of large load [35,36,37]. This is because the bond between FRP bars and concrete gradually loses with the increase of load, and the inhomogeneity coefficient of FRP bars increases gradually. In this paper, when the test beam is close to failure, the phenomenon of “false yield” mentioned above may even occur due to the large slip of FRP bars. It can be seen that the formula for calculating short-term flexural stiffness of FRP reinforced concrete beams needs further improvement.

## 5. The Modified Formula for Short Term Flexural Rigidity 

At present, there are big differences in the specifications or calculation models in different regions, and the factors considered in the formula are also different, which affect the accuracy of the calculation results of the fiber reinforced test beam. In addition, there are few studies on the stiffness of fiber reinforced coral concrete beams, and it is impossible to conduct a more comprehensive evaluation of its mechanical properties. Therefore, this article compares and analyzes the calculation results of different codes or models and proposes a stiffness calculation model for coral concrete beams on the basis of it.

### 5.1. Formula to Calculate ψ from Chinese Code (50010-2010)

According to the existing calculation theory of flexural rigidity of concrete beams, the elastic modulus of materials and the crack characteristics of members have a significant impact on the flexural behavior and stiffness calculation of concrete beams. For reinforced concrete beams with cracks, the concrete segment between cracks actually bears part of the tension stress, and the tensile strain of the longitudinal bar in the pure bending sector is not uniform. Therefore, the determination of strain inhomogeneity coefficient of longitudinal tension bars between cracks is very important for the stiffness calculation. Previous studies have shown that the tensile strength of concrete is almost proportional to the bond strength. Most of the formulas are fully expressed about the important influence of concrete tensile strength and actual reinforcement ratio on the inhomogeneity coefficient of reinforced concrete beams, which is in accordance with the actual stress state. The inhomogeneity coefficient ψ of steel strain in GB50010-2010 [38] is as follows:

(8) $$\psi =1.1(1-0.59\frac{f_{tk}}{\rho_{te}\sigma_{s}})=1.1-0.65\frac{f_{tk}}{\rho_{te}\sigma_{s}}$$

Obviously, the surface hardness and shear strength of steel bars are much better than that of FRP bars, and the shear failure of concrete is the main manifestation of interfacial slip. When the reinforcement ratio and the stress of steel bars are fixed, the inhomogeneity coefficient in Equation (8) decreases gradually with the increase of the tensile strength of concrete, which is in line with the actual situation. However, the bond-slip failure mechanism between FRP bars and concrete is more complex, and the bond performance between FRP bars and concrete is significantly different [23,24,25,26]. When the bond interface between FRP bars and concrete slips, not only the concrete is damaged by longitudinal shear, but also the surface of FRP bars [31]. When the strength of concrete increases to a certain extent, the damage focuses on the surface of FRP bars. Therefore, when the strength of concrete exceeds a certain range, the bond strength of concrete is only slightly improved with the increase of strength. There is no doubt that the inhomogeneity strain coefficient of FRP bars in flexural members will not keep a linear relationship with the tensile strength of concrete. The derived formula based on the linear relationship cannot correctly reflect the actual stress situation, especially when the concrete strength is high. 

**Figure 11:**
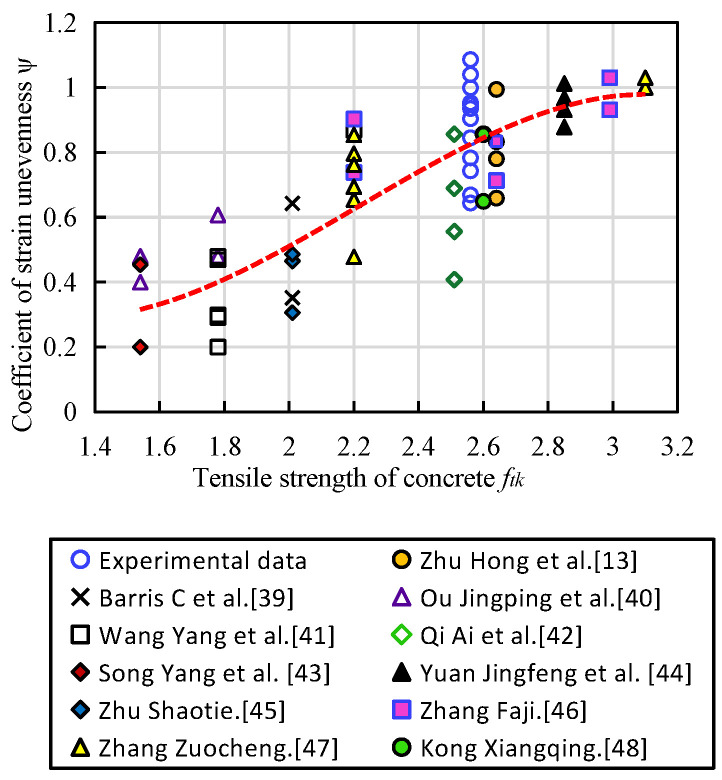
Relationship between tensile strength and strain inhomogeneity coefficient of concrete [14].

Since all the FRP bars of the flexural members have non-uniform strain and all change in a close range, in this paper, forty eight test results of simply supported concrete beams with CFRP, BFRP and GFRP bars of different strengths are investigated based on the experimental results and the available reference [13,39,40,41,42,43,44,45,46,47,48], and the relationships between the concrete tensile strength and the inhomogeneity coefficient is developed based on these results and is shown in Figure 11 [14]. The strain inhomogeneity coefficient of longitudinal reinforcement between cracks are derived into following formula:

(9) $$\psi =1.1-0.45\frac{f_{tk}\exp\frac{1}{5}(1.54-f_{tk})}{\rho_{te}\sigma_{fk}}$$

### 5.2. The Modified Formula for Flexural Rigidity 

When developing the modified short-term stiffness calculation formula of FRP bars reinforced coral concrete beams, two key factors need to be considered. Firstly, the influence of material properties and surface conditions of FRP bars on flexural stiffness should be considered. The influence factors of bonding performance of different FRP bars to coral concrete should be introduced. Based on the experimental data and the bonding characteristic coefficients $\beta_{s}$ of reinforced concrete in the code, the bonding characteristic coefficients of reinforcing bars should be taken into account. The value $\beta_{s}$ is taken as 0.9, and the influencing factor K1 reflecting the surface of FRP is introduced. (The value of K1 is influenced by the diameter, height, spacing and rib type of FRP bars, which is quite complex, and needs further study, however it is conservative to takes as 0.9 here [27,28,30].) Secondly, considering the effect of low modulus of elasticity of CFRP bars and coral concrete, and the crack height of concrete beams is higher resulting lever arm is larger, and the lever arm η is taken as 0.9 from Chinese code GB50608-2010, therefore, for the short-term stiffness Formula (10), the coefficient before ψ should be taken as 1/0.9=1.11. 

Based on theoretical analysis and experimental data, the formulas for calculating the short-term flexural stiffness of CFRP reinforced coral concrete beams are presented as follows:

(10) $$B_{S}=\frac{K_{1}\beta_{s}E_{f}A_{f}h_{of}^{2}}{1.11\psi +0.2+\frac{6\alpha_{fE}\rho_{f}}{1+3.5\gamma_{f}^{\prime }}}$$

(11) $$\sigma_{fk}=\frac{M_{k}}{0.9A_{f}h_{of}}$$

where $\beta_{s}$ is the effect coefficient of bond property between FRP bars and coral concrete, $K_{1}$ is the effect factor of FRP apparent condition, and the other symbols have the same meaning as Formula (3).

### 5.3. Validation of the Modified Formula 

**Figure 12:**
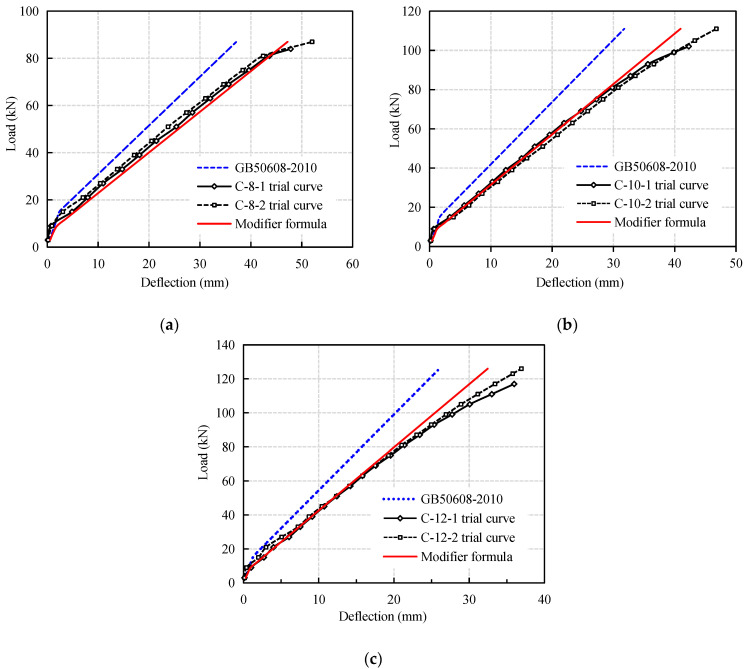
Comparison between result of the proposed formula and that of test results. (**a**) C-8; (**b**) C-10; (**c**) C-12.

Figure 12 is a comparison between the load-midspan deflection curves of CFRP reinforced coral concrete beams calculated by the modified formula and the test results. It can be seen that the calculated values are in good agreement with the experimental values within the range of service loads and tend to be safe. It is noteworthy that when approaching the ultimate load, the deflection of the test beam increases rapidly and destroys due to the larger slip of CFRP bars in coral concrete. Especially when it is close to destruction, which makes the calculation curve of the revised formula deviate to a certain extent, but the difference is still small. Further research is needed if the bending stiffness of the test beam is to be calculated more accurately when it approaches failure. 

### 5.4. Discussion

Analyzed from the test results, the existing calculation model tends to overestimate the flexural rigidity of the fiber reinforced coral concrete beam and have certain accuracy only before and after the cracking of the test beams. However, as the load increases, the error becomes larger, and the design requirements for different load levels cannot be met. In the case of test beam failure, the errors of Zhu et al. [13], GB50608-2010 [26], and ACI [48] are 17.1–20.8%, 27.6–28.7%, and 35–36% respectively. The model proposed in this paper has an error of no more than 3% within 80% of the working range, even if the error is only between 7% and 10% when it is broken.

In fact, the calculation model of fiber reinforcement basically refers to the reinforced concrete specification, and does not consider the apparent condition of the reinforcement, the relative slippage of the reinforcement and the influence of the force characteristics at different stages on the components, so it is impossible to accurately calculate the components. From another point of view, if one does not understand the force changes of the components at different loads or at different stages, when the components are damaged during use, they cannot be accurately evaluated and repaired. Therefore, in-depth study of stiffness changes has far-reaching significance for the application of fiber reinforced concrete members.

The calculation model proposed in this paper takes into account the force characteristics and influencing factors of the test beam at different stress stages and can predict the stiffness change and deflection deformation of the test beam under different loads. However, there is a lack of long-term mechanical performance test research on test beams and long-term load degradation and damage research in this field, so long-term stiffness prediction cannot be made. In the future work, Finite Element modelling [49] can be further performed, as well consider other parameters such as effect of high strength concrete [50] 

## 6. Conclusions

1. The flexural behavior of CFRP reinforced coral concrete beams is similar to that of steel reinforced concrete beams with good ductility, but the flexural stiffness of CFRP reinforced coral concrete beams is significantly lower than that of ordinary reinforced concrete beams. There is no obvious yield stage. After the cracking of concrete in tension zone, the stiffness of CFRP reinforced coral concrete beams decreases rapidly and the increase rate of deflection accelerates obviously.
2. Increasing the reinforcement ratio of CFRP bars in a certain range can significantly improve the flexural rigidity and reduce the deflection of CFRP reinforced coral concrete beams. However, by increasing the reinforcement ratio to reduce the deflection of the test beam, it will reduce the utilization of the strength of the reinforcement.
3. The bond performance between CFRP bars and coral concrete has a significant impact on the flexural performance and failure mode of CFRP bars. Most of the tested beams show obvious slip phenomenon under tension, which results in serious loss of flexural capacity of the flexural-shear section.
4. The revised formula for strain inhomogeneity coefficient of CFRP bars in coral concrete beams proposed in this paper has higher accuracy.
5. A new short-term stiffness formula of CFRP reinforced coral concrete beams is developed, which considering the low elastic modulus of FRP bars, the stress-strain characteristics of coral concrete and the bond performance. The relative slip coefficient is taken as 0.9, and the surface condition coefficient is 0.9. The calculated results are close to the experimental values.

## Figures and Tables

**Table 1:** Mechanical properties of CFRP bars.

Designated Diameter (mm)	Minor Diameter (mm)	Rib Height (mm)	Rib Spacing (mm)	Ultimate Tensile Strength *f_fu_*(MPa)	Elastic Modulus *E_f_* (GPa)
8	7.62	0.44	8.65	1628.3 ± 16	106.4
10	9.21	0.18	8.78	1515.9 ± 20	108.6
12	11.08	0.53	8.93	1910.8 ± 27	111

**Table 2:** Basic mechanical properties of coral concrete.

Concrete Grade	Water-Cement Ratio	*f_c_* (MPa)		*f_cu_* (MPa)		*f_ct_* (MPa)		*E_c_* (GPa)	
		Mean	Error	Mean	Error	Mean	Error	Mean	Error
C45	0.28	46.4	±3.2	42.6	±2.4	2.56	±0.2	31.6	±1.1

NB: *f_cu_* is cylindric compressive strength.

**Table 3:** Parameters test beams.

Beam	N ^(1)^	Size (mm) (b × h × *l_0_*)	h_1_^(2)^	Longitudinal Bar	D ^(3)^ (mm)	Longitudinal Erecting Bar	Stirrup	Reinforcement Ratio (%)
C-8	2	120 × 250 × 2100	25	2 CFRP	8	2ϕ6(CFRP)	ϕ6 CFRP	0.37
C-10	2	120 × 250 × 2100	25	2 CFRP	10	2ϕ6(CFRP)	ϕ6 CFRP	0.58
C-12	2	120 × 250 × 2100	25	2 CFRP	12	2ϕ6(CFRP)	ϕ6 CFRP	0.84
S-14	1	120 × 250 × 2100	25	2 Steel bars	14	2ϕ6(CFRP)	ϕ6 CFRP	1.14
S-16	1	120 × 250 × 2100	25	2 steel bars	16	2ϕ6(CFRP)	ϕ6 CFRP	1.49

^(1)^ N = Number of test beams. ^(2)^ h_1_ = Protection thickness. ^(3)^ D = Diameter.

**Table 4:** Cracking and failure of test beams.

Beam	$\rho_{f}^{\left(1\right)}$ (%)	Cracking		Failure			Ultimate Strength (kN)	Number of Cracks	Failure Mode
		Load (kN)	$w^{\left(2\right)}$ (mm)	$w_{\max}^{\left(3\right)}$ (mm)	$w_{}^{\left(4\right)}$ (mm)	P^(5)^(*f* = 10.5 mm) (kN)			
C-8-1	0.37	9	0.13	2.45	1.33	26	84	13	Shear failure
C-8-2	0.37	9	0.13	1.85	1.11	28	87	15	CFRP bar rupture
C-10-1	0.58	9	0.13	1.81	1.41	34	102	13	CFRP bar slip
C-10-2	0.58	9	0.13	2.34	1.67	32	111	11	Concrete Crash
C-12-1	0.84	9	0.08	1.49	0.99	45	117	14	Concrete Crash
C-12-2	0.84	12	0.08	1.16	0.89	46	126	14	Concrete Crash
S-14-1	1.14	15	0.06	1.1	0.55	73	81	13	Concrete Crash
S-16-1	1.49	15	0.06	0.5	0.27	82	93	14	Concrete Crash

^(1)^$\rho_{f}$ = Reinforcement ratio. ^(2)^ w = Average crack width at time of cracking. ^(3)^ w = Maximum crack width when test beam is destroyed. ^(4)^ w = Average crack width when the test beam is broken. ^(5)^ P = When the deflection of the test beam reaches *l_0_*/200, the corresponding external load value.

## Data Availability

The data used to support the findings of this study are available from the authors upon request.

## References

[1] Da B., Yu H., Ma H., Tan Y., Mi R., Dou X. (2016). Chloride diffusion study of coral concrete in a marie environment. Constr. Build. Mater..

[2] Wang A.G., Lyu B.C., Zhang Z.H., Liu K.W., Xu H.Y., Sun D.S. (2018). The development of coral concretes and their upgrading technologies: A critical review. Constr. Build. Mater..

[3] Monaldo E., Nerilli F., Vairo G. (2019). Basalt-based fiber-reinforced materials and structural applications in civil engineering. Compos. Struct..

[4] Fergani H., Di Benedetti M., Oller C.M., Lynsdale C., Guadagnini M. (2018). Long-term performance of GFRP bars in concrete elements under sustained load and environmental actions. Compos. Struct..

[5] El-Nemr A., Ahmed E.A., Benmokrane B. (2013). Flexural behavior and serviceability of normal-and high-strength concrete beams reinforced with glass fiber-reinforced polymer bars. ACI Struct. J..

[6] Jiang J.F., Luo J., Yu J.T., Wang Z.C. (2019). Performance improvement of a fiber-reinforced polymer bar for a reinforced sea sand and seawater concrete beam in the serviceability limit state. Sensors.

[7] Grace N.F., Soliman A.K., Abdel-Sayed G., Saleh K.P. (1998). Behavior and ductility of simple and continuous FRP reinforced beams. J. Compos. Constr..

[8] Elgabbas F., Vincent P., Ahmed E.A., Benmokrane B. (2016). Experimental testing of basalt-fiber-reinforced polymer bars in concrete beams. Compos. Part B Eng..

[9] Kassem C., Farghaly A.S., Benmokrane B. (2011). Evaluation of flexural behavior and serviceability performance of concrete beams reinforced with FRP bars. J. Compos. Constr..

[10] Gdoutos E.E., Pilakoutas K., Rodopoulos C.A. (2000). Failure Analysis of Industrial Composite Materials.

[11] Solyom S., Balázs G.L. (2020). Bond of FRP bars with different surface characteristics. Constr. Build. Mater..

[12] Wang L., Shen N., Zhang M., Qian K., Fu F. (2020). Bond performance of Steel-CFRP Bar reinforced Coral Concrete beams. Constr. Build. Mater..

[13] Hong Z., Zhiqiang D., Gang W., Zhisheng W. (2015). Experimental study and theoretical calculation on the flexural stiffness of concrete beams reinforced with FRP bars. China Civ. Eng. J..

[14] Wang L., Zhang J., Chen W., Fu F., Qian K. (2020). Short term Crack width Prediction of CFRP Bars reinforced Coral Concrete. Eng. Struct..

[15] Issa M.S., Metwally I.M., Elzeiny S.M. (2011). Influence of fibers on flexural behavior and ductility of concrete beams reinforced with GFRP rebars. Eng. Struct..

[16] Gravina R.J., Smith S.T. (2008). Flexural behaviour of indeterminate concrete beams reinforced with FRP bars. Eng. Struct..

[17] Al-Sunna R., Pilakoutas K., Hajirasouliha I., Guadagnini M. (2012). Deflection behaviour of FRP reinforced concrete beams and slabs: An experimental investigation. Compos. Part B Eng..

[18] Wang L., Zhang J., Changshi H., Fu F. (2020). Comparative Study of Steel-FRP, FRP and Steel-Reinforced Coral Concrete Beams in Their Flexural Performance. Materials.

[19] Sun Z., Fu L., Feng D.-C., Vatuloka A.R. (2019). Experimental study on the flexural behavior of concrete beams reinforced with bundled hybrid steel/FRP bars. Eng. Struct..

[20] Sheng X.X., Tao J., Yongfeng Z. (2009). Characteristics and calculation methods of concrete beams reinforced with FRP bars. Eng. Mech..

[21] Gao S., Guo L.H., Fu F., Zhang S.M. (2017). Capacity of semi-rigid composite joints in accommodating column loss. J. Construct. Steel Res..

[22] Fu F., Lam D., Ye J. (2010). Moment resistance and rotation capacity of semi-rigid composite connections with precast hollowcore slabs. J. Construct. Steel Res..

[23] Guo L., Liu Y., Fu F., Huang H. (2019). Behavior of axially loaded circular stainless steel tube confined concrete stub columns. Thin Walled Struct..

[24] Qian K., Liang S.L., Xiong X.Y., Fu F., Fang Q. (2020). Quasi-static and dynamic behavior of precast concrete frames with high performance dry connections subjected to loss of a penultimate column scenario. Eng. Struct..

[25] Weng Y.H., Qian K., Fu F., Fang Q. (2020). Numerical investigation on load redistribution capacity of flat slab substructures to resist progressive collapse. J. Build. Eng..

[26] Chinese Standard (2011). GB50608-2010 Technical Code for Infrastructure Application of FRP Composites.

[27] Wei M.W., Xie J.H., Zhang H., Li J.L. (2019). Bond-slip behaviors of BFRP-to-concrete interfaces exposed to wet/dry cycles in chloride environment. Compos. Struct..

[28] Wang L., Mao Y., Lv H., Chen S., Li W. (2018). Bond properties between FRP bars and coral concrete under seawater conditions at 30, 60, and 80 degrees C. Constr. Build. Mater..

[29] Dong Z.Q., Wu G., Xu B., Wang X., Taerwe L. (2016). Bond durability of BFRP bars embedded in concrete under seawater conditions and the long-term bond strength prediction. Mater. Des..

[30] Gooranorimi O., Claure G., Suaris W., Nanni A. (2018). Bond-slip effect in flexural behavior of GFRP RC slabs. Compos. Struct..

[31] Achillides Z., Pilakoutas K. (2004). Bond behavior of fiber reinforced polymer bars under direct pullout conditions. J. Compos. Constr..

[32] ACI Committee 440 (2015). Guide for the Design and Construction of Concrete Reinforced with FRP Bars (ACI 440.1R-15).

[33] ACI Committee 440 (2013). Guide for the Design and Construction of Structural Concrete Reinforced with FRP Bars: ACI 440.1R-06.

[34] ACI Committee 318 (2005). Building Code Requirements for Structural Concrete (ACI318-05) and Commentary (318R-05).

[35] Atutis E., Valivonis J., Atutis M. (2018). Experimental study of concrete beams prestressed with basalt fiber reinforced polymers under cyclic load. Compos. Struct.

[36] Abdalla H.A. (2002). Evaluation of deflection in concrete members reinforced with Fibre Reinforced Polymer (FRP) Bars. Compos. Struct..

[37] Mias C., Torres L., Turon A., Barris C. (2013). Experimental study of immediate and time-dependent deflections of GFRP reinforced concrete beams. Compos. Struct..

[38] Chinese Standard (2011). GB50010-2010 Code for Design of Concrete Structures.

[39] Barris C., Torres L., Turon A., Baena M., Catalan A. (2009). An experimental study of the flexural behaviour of GFRP RC beams and comparison with prediction models. Compos. Struct..

[40] Jinping O., Bo W., Zheng H. (2005). Mechanical behavior of concrete beams reinforced with CFRP bars. China Civ. Eng..

[41] Wang Y., Dong H., Wang Z. (2008). Flexural behavior of concrete beams reinforced with GFRP bars. J. Harbin Inst. Tech..

[42] Qi A., Weng C. (2007). Experimental study on ductility behavior of concrete beams reinforced with FRP rebars. Earthq. Res. Eng. Retrofitt..

[43] Song Y., Zhang X., He M. (2014). Experimental research on flexural behavior of basalt FRP reinforeed concrete bearns. Eng. Plast. Appl..

[44] Yuan J. (2006). Analysis on Flexural Behavior of Concrete Beam Reinforced with FRP Bars.

[45] Zhu S. (2016). Experimental Study on Flexural Behavior of Concrete Beams Reinforced with BFRP Bars.

[46] Zhang F. (2018). Experimental Study on Flexural Behavior of High Performance Sea-Sand Concrete Beams Reinforced with FRP Bars.

[47] Zhang Z. (2005). Experimental Research and Mechanical Properties Analysis of Carbon Fiber Reinforced Concrete.

[48] Kong X., Yu Y., Xing L., Hang F., Liu H. (2018). Experimental study on flexural performance of BFRP bars and reinforced concrete beams with mixed reinforcement. FRP Composites.

[49] Fu F., Lam D., Ye J. (2008). Modelling semi-rigid composite joints with precast hollowcore slabs in hogging moment region. J. Constr. Steel Res..

[50] Deng X.-F., Liang S.-L., Fu F., Qian K. (2020). Effects of High-Strength Concrete on Progressive Collapse Resistance of Reinforced Concrete Frame. J. Struct. Eng..

